# Relaxation matters: comparison of in-vitro vasodilatory role of botulinum toxin-A and papaverine in human radial artery grafts

**DOI:** 10.1186/s13019-019-0837-7

**Published:** 2019-01-21

**Authors:** Omer Tanyeli, Ipek Duman, Yuksel Dereli, Niyazi Gormus, Hatice Toy, Ayse Saide Sahin

**Affiliations:** 1 Department of Cardiovascular Surgery, Meram Medicine Faculty, Necmettin Erbakan University, Konya, Turkey; 2 Department of Pharmacology, Meram Medicine Faculty, Necmettin Erbakan University, Konya, Turkey; 3 Department of Pathology, Meram Medicine Faculty, Necmettin Erbakan University, Konya, Turkey

**Keywords:** Botulinum toxin A, Botox, Papaverine, Radial artery, Vasodilation, Coronary artery bypass graft surgery

## Abstract

**Background:**

Radial artery (RA) is widely used in coronary artery bypass (CABG) surgery and the prevention of spasm is crucial for graft patency. Botulinum toxin A (BTX-A) and B are commonly used for aesthetic reasons and neuromuscular disorders. They are proven to raise blood flow and increase survival of ischemic skin flaps. In this study we evaluated and compared the vasodilator effects of BTX-A and papaverine on human RA grafts.

**Methods:**

After resting 60 min in isolated organ baths, human RA grafts were examined. Contraction responses for different doses of serotonin (5-HT) and endothelin-1 (ET-1) were evaluated as a percent of maximum contraction response elicited by 80 mM potassium chloride (KCl). The inhibitory effects of BTX-A and papaverine on contraction responses taken at the 0th hour were compared with the 1st and 2nd hour responses. Inhibitory effects of BTX-A and papaverine against the contractile agent were evaluated by comparing the results of the first and last (0th and 2nd hour) application.

**Results:**

In low concentrations, when we compared the effects of BTX-A (10^− 8^ M) and papaverine (10^− 6^ M) on 5-HT, papaverine was found to be more effective at both the 0th and 2nd hour (*p* < 0.05). Both BTX-A and papaverine inhibited the maximum contractile effect of ET-1 to the same extent at the 0th hour; but, the inhibitory effect of BTX-A was significantly stronger at the 2nd hour (*p* < 0.05).

In high concentrations, when we compared the effects of BTX-A (10^− 6^ M) and papaverine (10^− 4^ M) on 5-HT, papaverine showed stronger inhibition (p < 0.05), whereas both agents had similar action of inhibition on ET-1 mediated maximum contraction responses.

**Conclusion:**

BTX-A inhibits both ET-1 and 5-HT induced contractions and its effectiveness does not decrease over time as observed with papaverine. This study is the first in the literature using human RA for prevention of vasospasm by BTX-A.

## Background

CABG is the most common cardiac surgery performed worldwide since it is the most effective revascularization method for several categories of patients. The success of the surgery depends on the patency of the conduits used for bypassing the occluded coronary arteries. In fact, patency is the key factor for the success of the operation. Although several arterial and venous conduits have been proposed, only four have been accepted in routine clinical use: the Internal Mammarian artery (IMA), the RA, the Gastroepiploic artery (GEA), and the Great Saphenous vein (GSV) [1]. As arterial grafts are live conduits and tend to react to native competitive flow much more than venous grafts, a functional characterization of the target vessel is an important part of the procedure. The RA also has a high reactive potential to vasoconstriction. Various surgical techniques and pharmacologic agents have been proposed to overcome this problem. Unfortunately, there is no perfect vasodilator that is effective in every situation since vasospasm can have multiple causes. In the operating room papaverine (1 mg/mL, 2.7 mmol/L) is satisfactory for topical use. However, its onset is slow and its acidity restricts intraluminal use. Sodium nitroprusside (1.7 mmol/L, 0.5 mg/mL), used topically, is very potent and may cause hypotension if it enters the systemic circulation [2].

For the last two decades, BTX-A and BTX-B have been commonly used in the medical arena, especially for aesthetic reasons and neuromuscular disorders. In an in-vitro study, botulinum toxin was also shown to be effective for prevention of arterial graft spasm in samples of rat abdominal aorta [3]. A positive change in blood flow of the femoral arteries in rats was also observed after injection of BTX-A [4]. BTX-A increased the survival rate of random cutaneous flaps by means of selective suppression of the sympathetic neurons of the cutaneous microcirculation system [5]. Pretreatment with BTX-A was associated with a lower rate of arterial and venous thrombosis in a rabbit model microanastomosis [6].

The contraction of RA is a major problem and BTX-A appears to be a good agent for resolving this problem. In this study, we examined both vasodilator effects of BTX-A and papaverine on human RA grafts in conjunction with possible histopathological changes, with a great potential of use in our routine daily practice.

## Methods

After receiving approval from the Local Institutional Ethics Committee (Project number: 2016/494; March 18th, 2016) and written and signed informed consents from the patients, all experimental procedures were held in our Pharmacology and Pathology Research Laboratories.

**Fig. 1:**
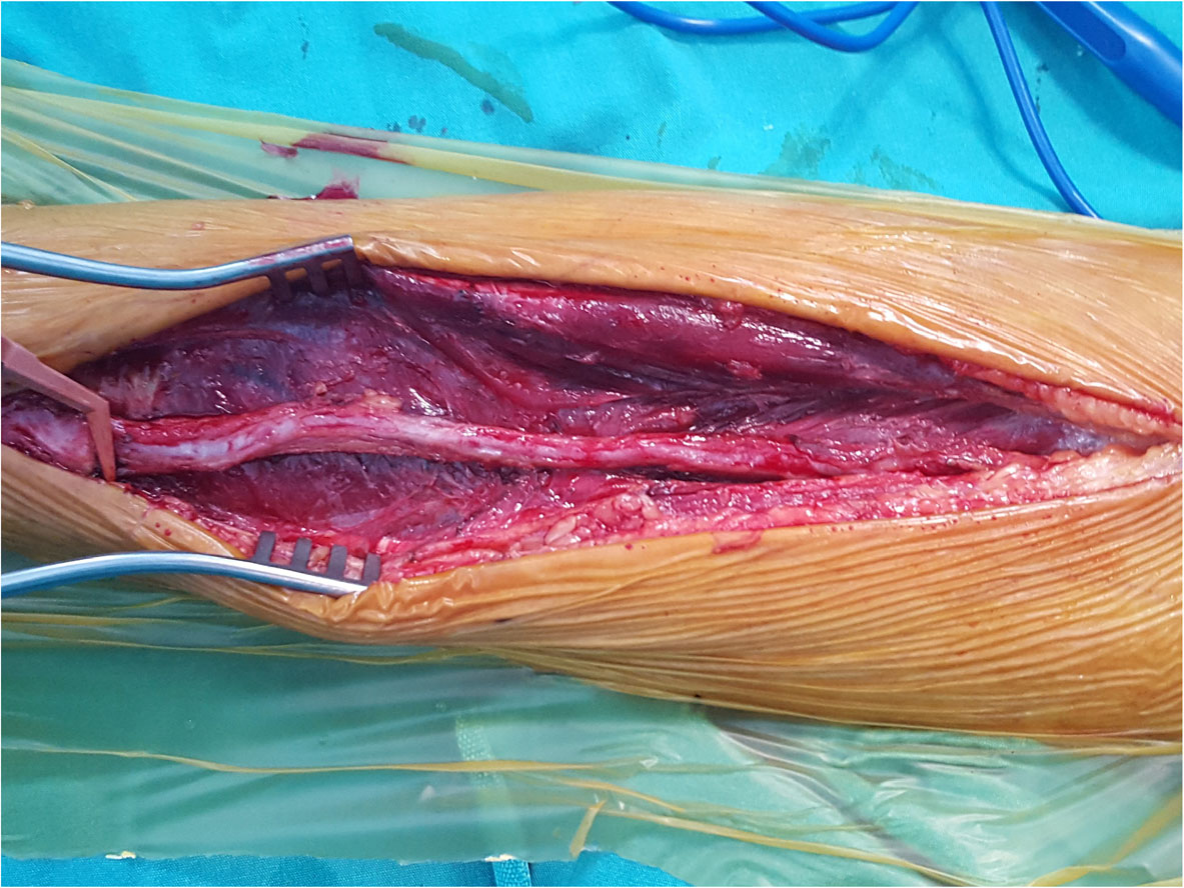
Operative view from radial artery harvesting. Note the fascia over the radial artery is peeled to prevent vasospasm

The patients in whom a RA graft would be used for CABG surgery were evaluated pre-operatively. Patients with a stenosis rate of at least 90% over the right coronary artery or circumflex artery and its’ obtuse marginal branches were potential candidates for RA harvesting. If the lesions were so, then a RA diameter of at least 2 mm over the wrist region with a negative Allen test were harvested from the patient’s non-dominant arm. The incision was started from the wrist using the no-touch technique and continued to the brachial artery bifurcation over the elbow region using a vessel sealer and divider (The LigaSure™ small jaw instrument, Covidien, Boulder, CO). The RA is first divided from the wrist and then from the elbow region. After controlling for bleeding, the RA is kept in papaverine impregnated gauze until it is used as graft. The operational procedure is shown in Fig. 1. During the operation, the RA distal anastomosis was made over the target stenotic coronary artery. After completing the distal anastomosis, the clamp over the aorta was removed and the heart began to beat. Next, the length of the RA and other grafts were measured and the proximal anastomosis was made over the ascending aorta with the aid of a side clamp. During this process, excess RA tissues obtained from non-diabetic male patients were used for our experimental procedures. Patients receiving calcium channel blocking agents and nitrates during the preoperative period were excluded from the study. Only arteries without macroscopic evidence of atherosclerosis were selected for tissue preparation.

These RA grafts were immediately sent to the Pharmacology Laboratory, being kept in Krebs-Henseleit solutions (KHS) ([mM] NaCl: 118.3; KCl: 4.69; KH_2_PO_4_: 1.18; CaCl_2_: 1.25; MgSO_4_: 1.17; NaHCO_3_: 25.0; Glucose: 11.1; pH: 7.4). The RA grafts were then cut into 2–3 mm circular slices. A total of 2–4 rings were obtained from each vessel segment (according to the length of tissue) and relaxation studies were conducted in different rings of the same vessel. The RA rings were randomly allocated to different experiment groups with sequentially numbered samples, to eliminate the influence of the drug therapy. In order to washout any residual drug effect, rings were repeatedly washed with KHS every 15 min for three hours, so that possible effects of papaverine during the harvest procedure were eliminated. The tissues were then transferred to isolated organ baths containing KHS and aired by 95% O_2_ and 5% CO_2_, which were kept at a constant 37 °C temperature and rested for another 60 min under 2 g of resting tension. Responses to the agents were recorded by a transducer (Biopac MP36, California, USA) isometrically (Commat, Ankara, Turkey).

The sample size was calculated using G*Power, version 3.1.9.2 (Heinrich Heine University, Dusseldorf, Germany) software package on computer. For the reasons of planning to use two-way repeated measures of ANOVA for data analysis, to conduct the trial on 10 groups with three repeated measures in each group, and to assume a correlation as 0.60 among the measures, the sample size was calculated to be a total of at least 60 for the trial, with an effect size of 25%, a power of 85%, and a confidence level of 95%. By allocating the total sample size to 60 rings in a 1:1 allocation ratio, 6 samples were included in each group.

### Experimental procedure

At the end of the resting period, maximum contraction responses were obtained by adding 80 mM KCl to the bath. Tissues were then rested by washing nutritious solutions. After 40 min of incubation with BTX-A (10^− 8^ M or 10^− 6^ M, Allergan Pharmaceuticals, Dublin, Ireland, prepared with distilled water) tissues were washed and cumulative concentration-response curves were obtained by ET-1 (10^− 12^–10^− 8^ M, Sigma Aldrich, St. Louis, MO, USA, stock solution prepared with 1% acetic acid) or 5-HT (10^− 9^–10^− 6^ M, Sigma Aldrich, St. Louis, MO, USA, prepared with distilled water). This application was considered to be the 0th hour. After taking maximum contraction responses, the tissues were washed again by nutritious solutions and dose-responses curves were repeated at the 1st and 2nd hours using the same contractive agents. In each tissue, only one of these contractive agents was tested. The same procedure was also repeated using papaverine (10^− 6^ M or 10^− 4^ M) after 40 min of incubation.

To compare the effect of incubation with BTX-A and papaverine, cumulative concentration-response curves were obtained with ET-1 (10^− 12^–10^− 8^ M) and 5-HT (10^− 9^–10^− 6^ M) at 0th, 1st and 2nd hours in two other groups of rings (*n* = 6). The responses to the contractive agents were evaluated as a percent (%) of the responses to KCl. The results of the study were calculated as mean ± standard deviation (SD).

In order to determine the time related course of contraction responses to BTX-A or papaverine (Sigma Aldrich, St. Louis, MO, USA, prepared with distilled water) induced inhibition, contraction responses at the 0th hour were compared with the responses at the end of the 1st and 2nd hours after incubation of these agents at each dose.

**Fig. 2:**
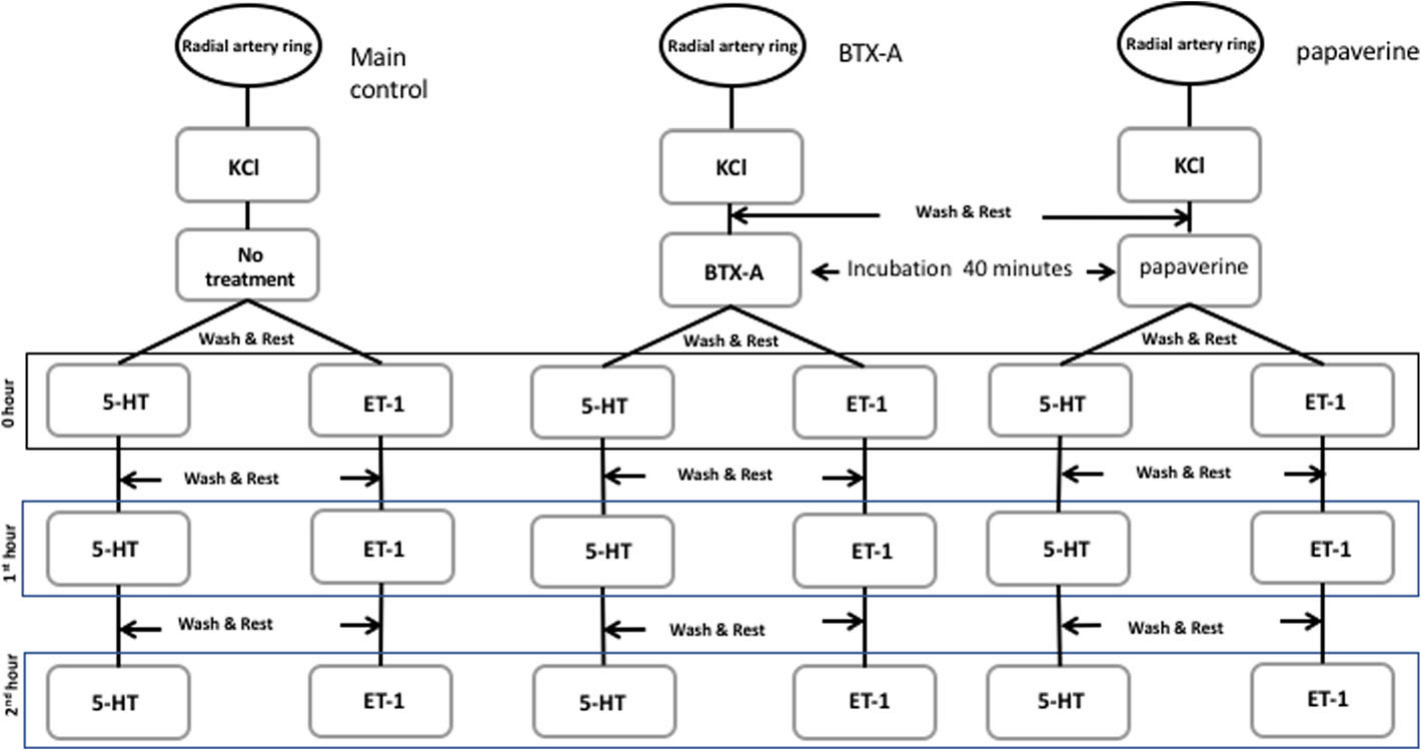
Flow-chart of the experimental procedures. KCL: potassium chloride (80 mM), BTX-A: botulinum toxin A (10^− 8^ M or 10^− 6^ M), papaverine (10^− 6^ M or 10^− 4^ M), cumulative 5-HT (10^− 9^ M - 10^− 6^ M), cumulative ET-1 (10^− 12^ M - 10^− 8^ M)

In addition to these comparisons, changes in the effects of BTX-A and papaverine by contractive agents was evaluated by comparing the first (0th hour) and the last (2nd hour) application. To this end, only the maximum contraction responses obtained by the maximum 5-HT and ET-1 concentrations were analyzed after incubation with BTX-A or papaverine. These responses were expressed as percent of the maximum contraction responses obtained by 5-HT or ET-1 in non-incubated tissues. These values were used to; (a) compare the inhibitory effects of BTX-A and papaverine, and (b) evaluate the effects of BTX-A and papaverine over the maximum contraction responses according to the changes obtained by contractive agents. A flow chart summarizing the experimental procedure is shown in Fig. 2.

At the end of the study, tissues were also evaluated by histopathological examination for endothelial tissue integrity. All samples were sent to the pathology laboratory in a 10% formaldehyde solution. After routine tissue preparation and blocking procedures, 5 μm thick slices were obtained. They were stained with hematoxylene and eosine (H&E) and all of the vascular layers (intima, media and adventitia) were examined under light microscope for fibrosis, necrosis, and any other pathological changes.

Statistical analysis was performed using a computer software package [(SPSS Statistics, version 17.0 (SPSS Inc., Chicago, IL, USA)]. The data were expressed as mean ± SD. A two-way repeated measures of ANOVA were used to compare the repeated contraction responses by time and by groups. When statistically significant differences were found, Bonferroni-corrected post-hoc tests were used to identify the origin of the differences. An independent samples t-test was performed for comparing standardized contraction responses for the BTX-A and papaverine groups. Results were considered significant if *p* < 0,05 (if p < 0,025 with Bonferroni correction).

## Results

In our experiment, a total of 19 RA samples were used from the patients. Mean age of the patients were 53.8 years (age ranged between 32 and 70 years). The contraction responses obtained by ET-1 and 5-HT were repeatable and did not show time dependent changes.

### The effects of BTX-A over the contraction responses of 5-HT and ET-1 contraction responses obtained by 5-HT

In tissues incubated with 10^− 8^ M BTX-A (*n* = 6), significant inhibition was detected in contraction responses obtained by high doses of 5-HT concentrations (10^− 7^–10^− 6^ M) (*p* < 0.05). With these dose regimens, no time dependent changes were found in BTX-A induced inhibition values except with 10^− 8^ M 5-HT contractions in which significant inhibition was seen only at the end of the 2nd hour.

**Fig. 3:**
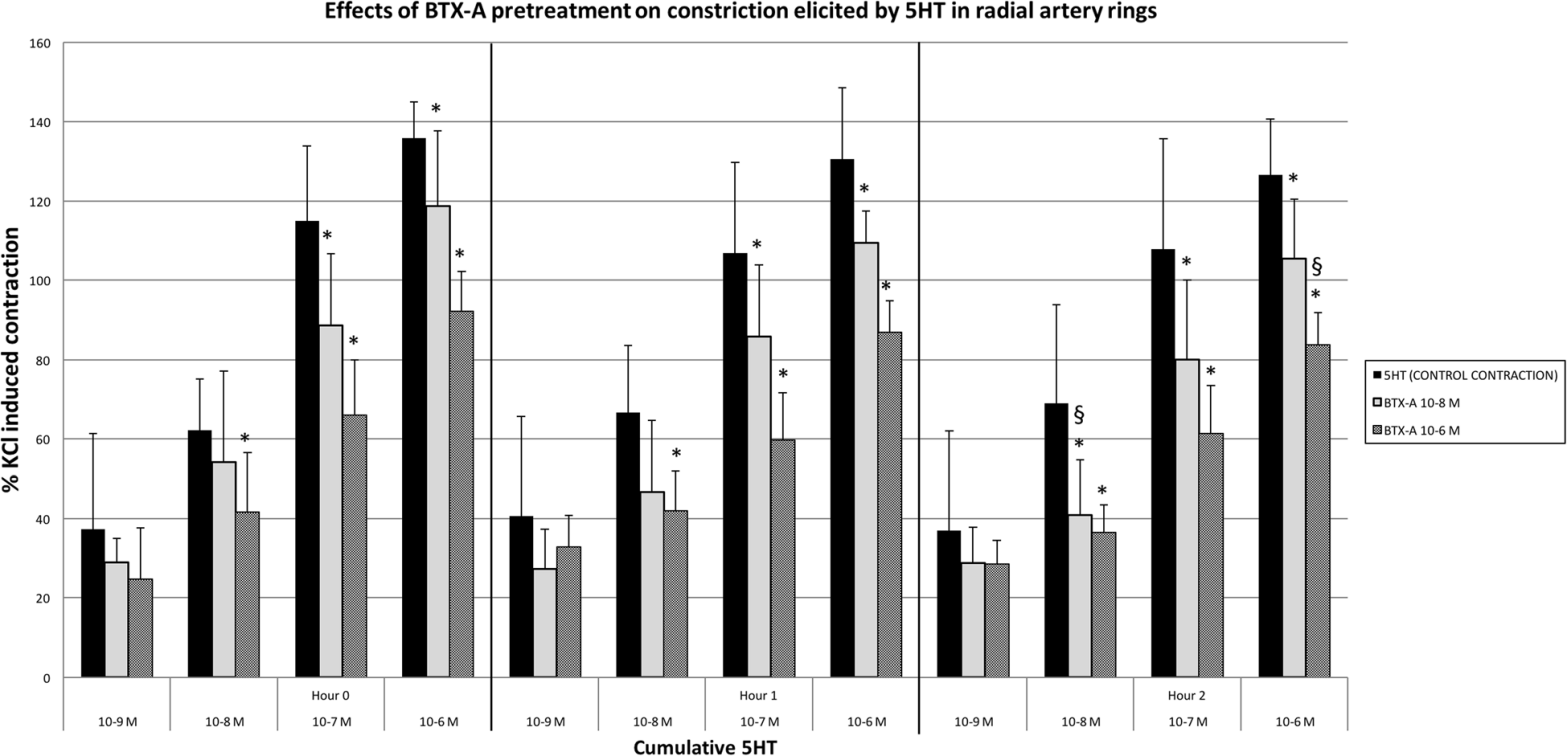
The effects of BTX-A pretreatment over the contraction responses of cumulative 5-HT. Results are % of KCl induced contractions, mean ± SD. *: *p* < 0.05 compared to control of each constrictor agent. ^**§**^: *p* < 0.05 increas in inhibition, compared to 0th hour

BTX-A of 10^− 6^ M (n = 6), inhibited all contraction responses in 5-HT concentrations (10^− 8^–10^− 6^ M), except for the lowest concentration (10^− 9^ M) in RA slices (*p* < 0.05). When the inhibitory concentrations of 5-HT were evaluated by time, only the inhibition at the highest concentration (10^− 6^ M) at the end of the 2nd hour increased when compared to other hours (*p* < 0.05). The inhibitory effects obtained by 10^− 8^ and 10^− 7^ M 5-HT contractions did not show either any increase or decrease by time (Fig. 3).

### Contraction responses obtained by ET-1

BTX-A of 10^− 8^ M (*n* = 6) significantly inhibited all initial contraction responses except at a concentration of 10^− 12^ M of ET-1 (*p* < 0.05), and it was shown that this inhibition increased at 1st and 2nd hours (p < 0.05). Same concentration of BTX-A (of 10^− 8^ M) inhibited ET-1 10^− 12^ M elicited contractions at the 2nd hour, while the initial and 1st hours responses were unaffected.

**Fig. 4:**
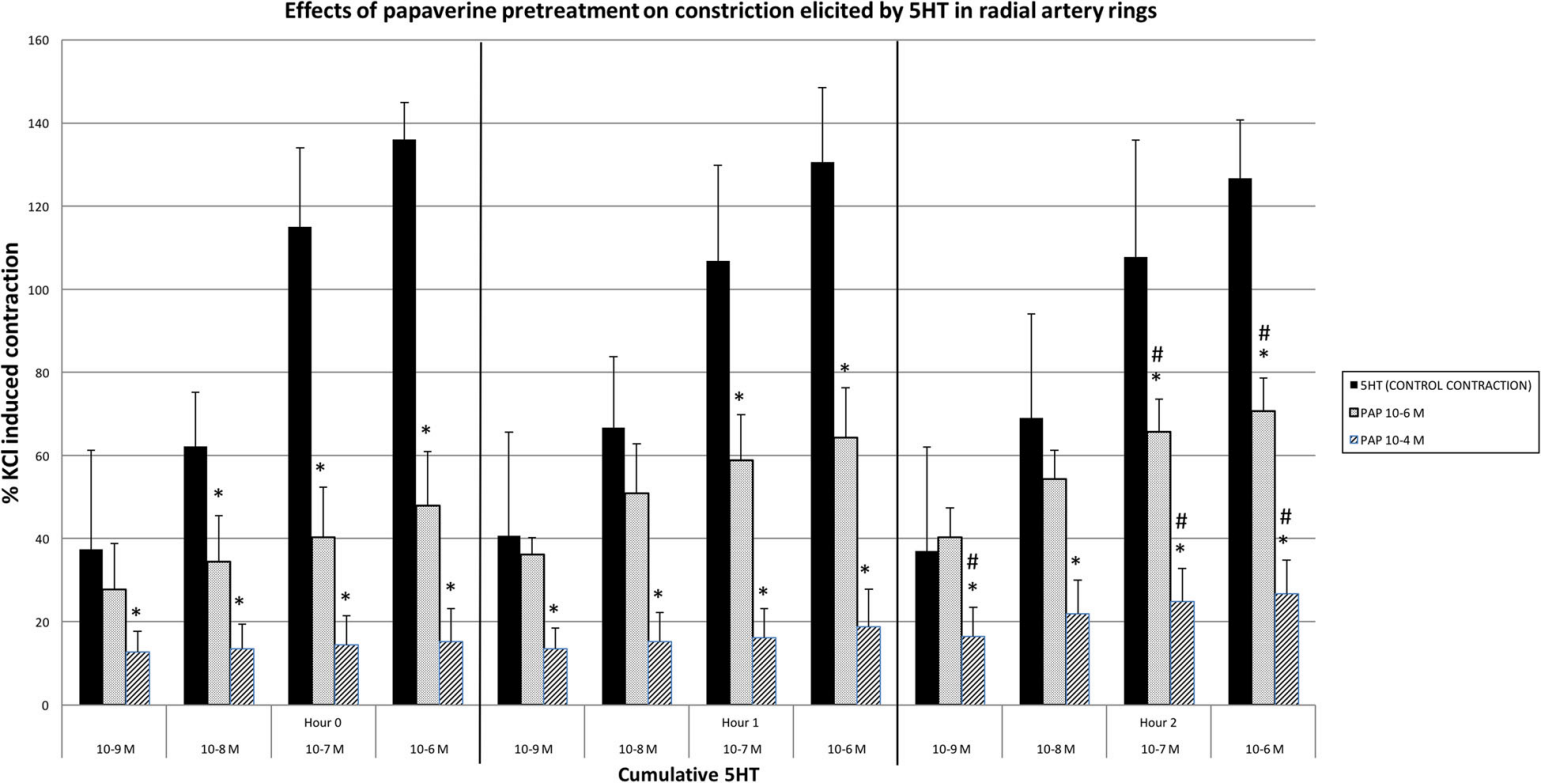
The effects of BTX-A pretreatment over the contraction responses of cumulative ET-1. Results are % of KCl induced contractions, mean ± SD. *: p < 0.05 compared to control of each constrictor agent. ^**§**^**:** *p* < 0.05 increase in inhibition, compared to 0th hour

Incubation with 10^− 6^ M BTX-A (*n* = 6) inhibited contraction responses at all ET-1 concentrations (p < 0.05). Inhibition of contraction responses obtained by 10^− 12^–10^− 11^ M ET-1 increased at 1st and 2nd hours (*p* < 0.05). Inhibition obtained by BTX-A at higher (10^− 10^–10^− 8^ M) ET-1 concentrations’ contraction responses did not change during the experiment (Fig. 4).

### The effects of papaverine over the contraction responses of 5-HT and ET-1 contraction responses obtained by 5-HT

Incubation with 10^− 6^ M papaverine (*n* = 6), inhibited the contraction responses at all concentrations, other than 10^− 9^ M 5-HT in the RA slices. This inhibitory effect decreased significantly at the 2nd hour (*p* < 0.05).

**Fig. 5:**
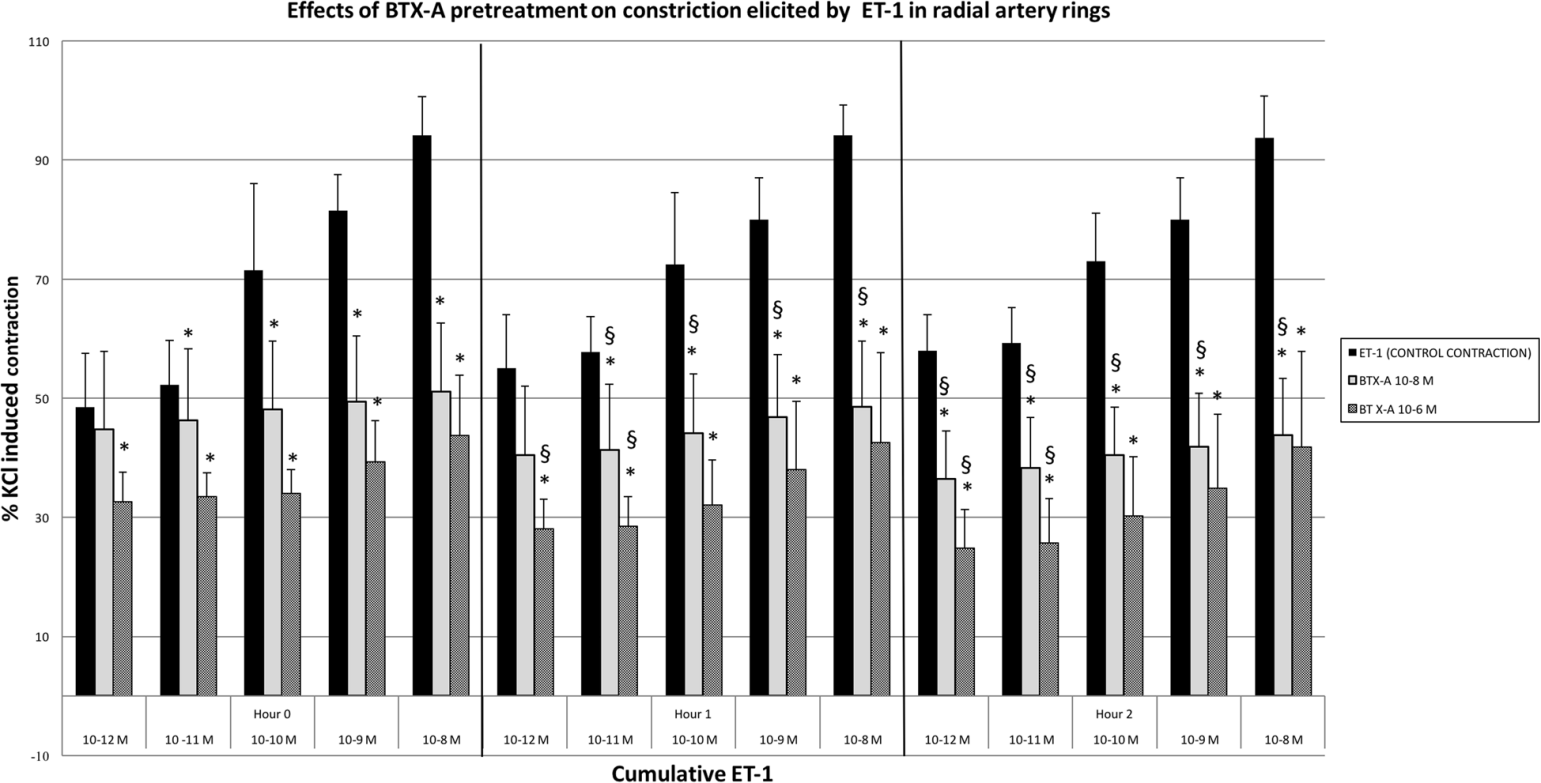
The effects of Papaverine pretreatment over the contraction responses of cumulative 5-HT. Results are % of KCl induced contractions, mean ± SD. *: *p* < 0.05 compared to control of each constrictor agent. ^#^: *p* < 0.05 decrease in inhibition compared to 0th hour

Incubation with 10^− 4^ M of papaverine (*n* = 6) significantly inhibited contractions in all 5-HT concentrations (*p* < 0.05). This inhibitory effect significantly decreased or even ceased at the 2nd hour in all 5-HT concentrations (p < 0.05) (Fig. 5).

### Contraction responses obtained by ET-1

In RA slices incubated with 10^− 6^ M papaverine (n = 6), significant inhibition was obtained only in the higher concentrations of ET-1 (10^− 9^–10^− 8^ M) mediated contraction responses and this inhibition significantly decreased both in the 1st and 2nd hours (*p* < 0.05).

**Fig. 6:**
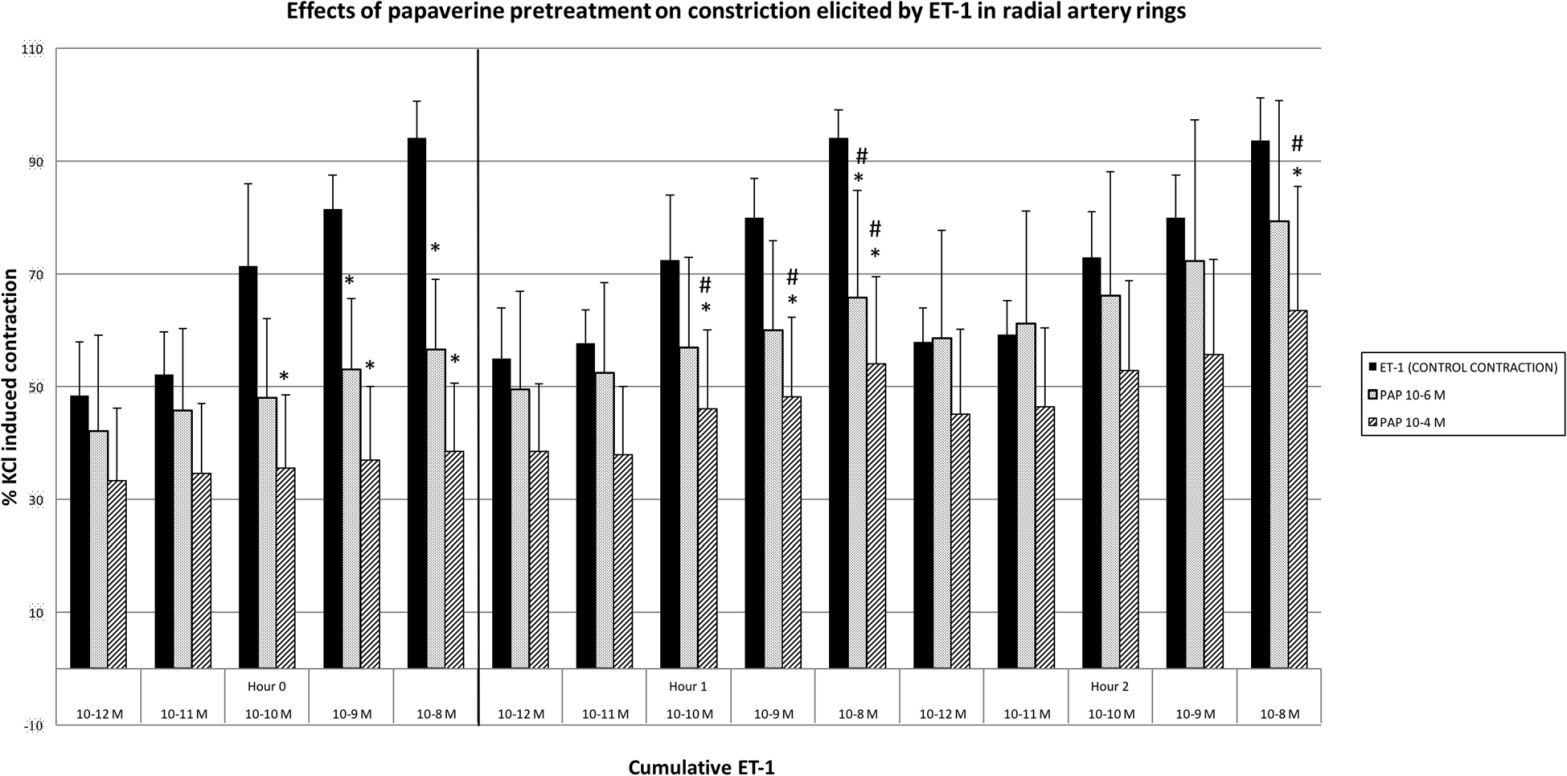
The effects of Papaverine pretreatment over the contraction responses of cumulative ET-1. Results are % of KCl induced contractions, mean ± SD. *: p < 0.05 compared to control of each constrictor agent. ^#^: *p* < 0.05 decrease in inhibition compared to 0th hour

Incubation with 10^− 4^ M papaverine (n = 6) inhibited ET-1 contractions at 10^− 10^–10^− 8^ M ET-1 concentrations. This inhibition decreased at the 1st and 2nd hours with these concentrations (p < 0.05) (Fig. 6).

### Comparison of inhibitory effects of BTX-A and papaverine

When comparing the effects of low concentrations of BTX-A (10^− 8^ M) and papaverine (10^− 6^ M) over 5-HT, it was observed that papaverine was more effective at both the 0th and 2nd hours (*p* < 0.05). Although both of the agents similarly inhibited the ET-1 mediated maximum contraction responses at the 0th hour, BTX-A inhibition was stronger at the 2nd hour (p < 0.05). (Table 1).

**Table 1:** Low dose inhibitors (Comparison of low dose BTX-A and papaverine)

Vasoconstrictor	Inhibitor agent	0th hour	2nd hour
5-HT 10^− 6^ M	BTX-A 10^− 8^ M	87.3 ± 11.1%	80.0 ± 7.1%
	Pap 10^− 6^ M	**35.2 ± 10.1%** ^*^	**51.9 ± 6.3%** ^*^
ET-1 10^− 8^ M	BTX-A 10^− 8^ M	54.3 ± 12.3%	46.5 ± 10.4%
	Pap 10^− 6^ M	61.0 ± 14.2%	**84.2 ± 23.1%** ^*^

5-HT: Serotonin, BTX-A: Botulium toxin A, ET-1: Endothelin 1, Pap: Papaverine. Results are % of vasoconstrictor (5-HT or ET-1) elicited maximal control contractions (Mean ± SD). *p < 0.05 compared to same hour BTX-A 10^− 8^ M (boldface entries)

When compared over contractions at high doses of BTX-A (10^− 6^ M) and papaverine (10^− 4^ M), it was shown that papaverine provided stronger inhibition over 5-HT mediated maximum contraction responses, but that both relaxing agents similarly inhibited ET-1 mediated maximum contraction responses (p < 0.05). (Table 2).

**Table 2:** High dose inhibitors (Comparison of high dose BTX-A and papaverine)

Vasoconstrictor	Inhibitor agent	0th hour	2nd hour
5-HT 10^− 6^ M	BTX-A 10^− 6^ M	67.8 ± 7.5%	61.6 ± 6.3%
	Pap 10^− 4^ M	**11.3 ± 6.1%** ^*^	**19.7 ± 6.4%** ^*^
ET-1 10^− 8^ M	BTX-A 10^− 6^ M	46.5 ± 10.7%	44.4 ± 16.8%
	Pap 10^− 4^ M	41.0 ± 13.2%	67.4 ± 23.7%

5-HT: Serotonin, BTX-A: Botulium toxin A, ET-1: Endothelin 1, Pap: Papaverine. Results are % of vasoconstrictor (5-HT or ET-1) elicited maximal control contractions (Mean ± SD). ^*^p < 0.05 compared to same hour BTX-A 10^−6^ M (boldface entries)

### Alteration of BTX-A and papaverine effects by contracting agents

Both BTX-A and papaverine inhibits contraction in radial artery strips depending on contractile agent (Tables 1, 2, and Figs. 2-5).

- 5-HT mediated maximum contraction responses: Although the inhibitory effects of both concentrations of papaverine on 5-HT elicited maximum contractions were higher than the inhibition caused by BTX-A, this inhibition showed significant time dependent reduction.
  The inhibition caused by lower concentration of BTX-A on the maximum 5-HT induced contraction did not show time dependent changes; but contrary to papaverine, the inhibitory effect was increased with time at higher concentration of BTX-A.
- ET-1 mediated maximum contraction responses: The effects of lower concentrations of BTX-A and papaverine on ET-1 elicited maximum contractions at 0th hour were similar. BTX-A resulted in higher inhibition at 2nd hour. Inhibition caused by papaverine decreased at 2nd hour.
  The effects of higher concentrations of BTA and PAP at both 0th and 2nd hour were similar. Inhibition caused by papaverine decreased at 2nd hour.

These findings illustrate that, the relaxing effects of BTX-A and papaverine change according to contracting agents. Although the inhibitory effect of papaverine significantly decreased by time in both concentrations over 5-HT mediated maximum contraction responses, it was stronger than the BTX-A mediated inhibitory effect. Low concentrations of BTX-A mediated inhibition did not change by time over 5-HT mediated maximum contractions; in high concentrations this effect increased by time, which is the opposite of the pattern observed with papaverine. In low concentrations, the inhibitory effects of BTX-A and papaverine were similar on ET-1 mediated maximum contractions at the first application; however at the last application BTX-A showed stronger inhibition than papaverine.

**Fig. 7:**
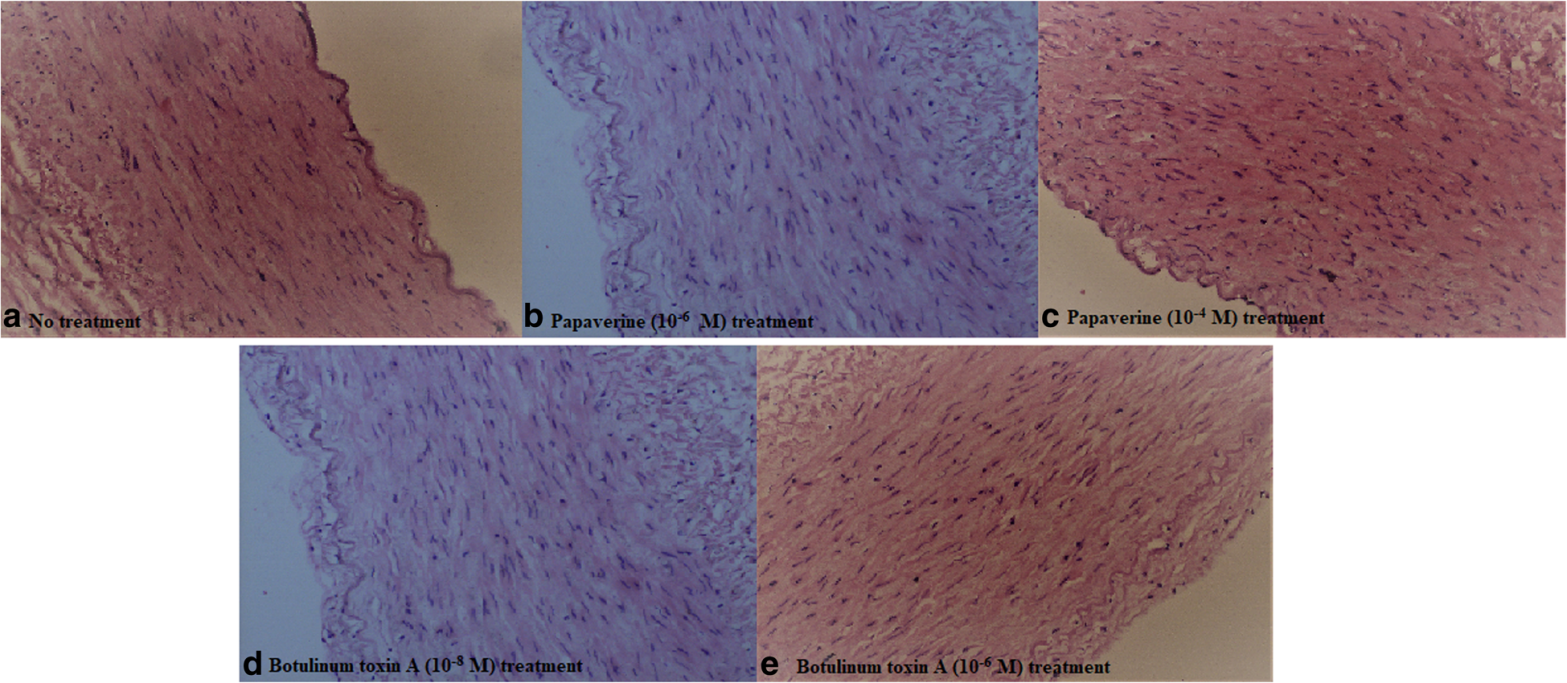
Histopathologic evaluation of papaverine (**b**-**c**) and Botulinum Toxin-A (**d**-**e**), made by Hematoxylene&Eosine stain under light microscopy (× 40 magnification) showed similar findings with the control group (**a**)

Histopathologic evaluation made by H&E stain under light microscopy showed similar findings with the control group. All of the vascular layers (intima, media and adventitia) kept their integrity. All of the samples in each group were evaluated for inflammation, fibrosis, necrosis, or mitotic activity. Histopathologically, none of the samples had fibrosis, inflammation, mitosis, or necrosis (Fig. 7).

## Discussion

Results of this in vitro study show that pretreatment with both papaverine and BTX-A prevents vasospasm of human RA rings. The duration of the inhibition of vasoconstriction elicited by potent vasoconstrictors was longer with BTX-A. No signs of endothelial damage were observed with the concentrations and durations of BTX-A (and also papaverine) used in this study.

Papaverine is widely used to prevent vasospasm in CABG surgery during graft preparation prior to implantation. It is a non-selective phosphodiesterase inhibitor found in the opium poppy [7]. It has been shown to increase cGMP and cAMP in smooth muscle, both of which induce vasorelaxation [8]. The use of papaverine is almost routine in surgical practice, but it has limited antispasmodic efficacy. It was shown to prevent vasoconstriction in response to potassium (60 mmol/L) and phenylephrine for only 1 h at the longest [9]. In our study, as a surgical procedure the RA grafts were kept in papaverine impregnated gauzes until the time they were anastomosed. After the anastomosis, the RA remnant tissues were sent to laboratory in KHS solution, and the tissues were washed to eliminate the possible residual effects. At least, 3-to-4 h passed until the first experimental procedure; that’s why, the possible effect of papaverine can be negligible in the experimental procedure.

BTX is a potent neurotoxin that is produced by the gram-positive, spore-forming, anaerobic bacterium, *Clostridium botulinum*. There are 7 known immunologically distinct serotypes of BTX: types A, B, C1, D, E, F and G [10]. Only Serotypes A and B are commercially available [11]. Although these seven neurotoxins are serologically distinct, they possess similar molecular weights and they have a common structure [12]. Serotype A appears to be the most potent subtype [13]. BTX-A inhibits muscle contraction by preventing the release of acetylcholine from the nerve terminal. The toxin is taken up by the nerve terminal and acts by blocking the transport of vesicles that contain acetylcholine [14]. BTX-A specifically binds and cleaves a protein, called synaptosomal associated protein 25 (SNAP 25). By inhibiting SNAP 25, vesicles with acetylcholine no longer migrate to the nerve terminal membrane; therefore the muscle does not contract and is rendered paralyzed [15]. Two additional mechanisms affect vasoconstriction: First, it blocks the transmission of the norepinephrine vesicle, preventing sympathetic vasoconstriction of the vascular smooth muscle [16]. Second, it blocks recruitment of the specific α2-adrenoceptor, which decreases the activity of chronically upregulated C-fiber nociceptors [17]. BTX interacts with the Rho/Rho kinase. BTX inactivates Rho kinase and inhibits smooth muscle cell constriction directly through interference with the Ca^2+^ sensitivity of vascular smooth muscle cells and the NO system [18].

Through the mechanisms explained above, BTX has several clinical applications, primarily in plastic surgery, neurology, and ophthalmology. The FDA has approved BTX for uses including moderate to severe glabellar lines, hyperhidrosis, blepharospasm, ocular strabismus, cervical dystonia, and torticollis [19]. In the last decade, new therapeutic options for BTX have expanded physicians’ minds, especially in the vascular field. Hayashi et al. documented a partial increase in blood flow in the femoral artery in rats [4]. Fathi et al. showed that pre-treatment with BTX-A was associated with a significantly larger arterial and venous diameter before microanastomosis. Moreover, BTX-A was also associated with a lower rate of arterial and venous thrombosis after microanastomosis [6]. In an experimental model, papaverine was compared with BTX-A for prevention of arterial graft spasm in rats’ abdominal aortas in the presence of KCl and noradrenaline. Almost all concentrations of BTX-A completely inhibited arterial contractions when compared with controls. BTX-A had a longer lasting effect than papaverine, with no toxic effect on the artery [3]. Schweiser et al. showed on a vascular, tissue, cell, and molecular level that BTX injection to the feeding arteries supports flap survival through ameliorated blood flow and oxygen delivery [20]. Besides laboratory use, clinical uses in the vascular field have increased. For example, BTX-A has received increasing attention as a means of relieving symptoms of vasospasm in Raynaud’s phenomenon. BTX-A is thought to function by blocking vascular smooth muscle depolarization and vasoconstriction, in addition to blocking of central nervous stimuli for vasoconstriction [21].

Our study is the first in the literature using human RA for prevention of vasospasm by BTX-A. As stated above, vasospasm of the RA remains one of the biggest problems in cardiac surgery. The high patency rates of RA make it the first choice graft in arterial draft selection after IMA. When compared with the routinely used vasodilator effect of papaverine, BTX-A showed encouraging results in our study. BTX-A inhibits ET-1 and 5-HT induced contractions. This inhibition differed according to the vasoconstrictor agent and, contrary to papaverine, did not decrease with time. The most important point in this study is probably the long-lasting effect of BTX-A on inhibition of vasoconstriction. We know from the clinical use of BTX that its effects can last up to 6 months. In clinical practice, this may lead to increased and long-lasting vasodilation of BTX-A treated RA grafts in CABG operations, where the grafts are more prone to occlusion in the beginning of the first month.

### Limitations

Limitation of this study is to conduct the experimental procedure with limited number of patients, since only the remnants of RA grafts were used. In-vitro analysis may have different consequences when compared to in-vivo studies. Nevertheless, we think that, human RA behavior will not change so much in-vivo.

## Conclusion

In conclusion, in-vitro pretreatment with BTX-A and papaverine is effective and safe in preventing vasospasm of RA graft conduits in the early period. The longer duration of BTX-A seems to be an additional advantage over papaverine. Further research is needed to assess the clinical results of BTX-A including the intra-luminal administration of BTX-A into clamped RAs in-vivo.

## Abbreviations

5-HT: Serotonin
BTX: Botulinum toxin
BTX-A: Botulinum toxin A
BTX-B: Botulinum toxin B
CABG: Coronary artery bypass
ET-1: Endothelin-1
GEA: Gastroepiploic artery
GSV: Great saphenous vein
H&E: Hematoxylene and eosine
IMA: Internal mammarian artery
KCl: Potassium chloride
KHS: Krebs-Henseleit solution
RA: Radial artery
SNAP: Synaptosomal associated protein
